# Efficacy of Intraoperative Periarticular Local Infiltration for Pain Control and Ambulation in Total Knee Arthroplasty: A Randomized Case-Control Study

**DOI:** 10.7759/cureus.52639

**Published:** 2024-01-20

**Authors:** Gopalakrishna KG, Rakshit J

**Affiliations:** 1 Department of Orthopedics, Bangalore Medical College and Research Institute, Bengaluru, IND

**Keywords:** additional analgesia, visual analogue scale, cocktail injection, intraoperative, total knee replacement

## Abstract

Introduction: Total knee arthroplasty (TKA) is a leading operative procedure for late-stage knee osteoarthritis. The cornerstone of a successful TKA is swift and effective rehabilitation to achieve a pain-free and good range of motion. Pain post-replacement hinders an effective rehabilitation protocol. Reported preoperative, perioperative, and postoperative analgesia modes have undesirable side effects. The purpose of this study is to assess the effect of a unique cocktail injection on immediate postoperative pain using the visual analog score, the need for additional analgesics during the initial period, and the ambulation time between the case and control groups.

Materials and methods: In this randomized case-control study, the periarticular injection consisted of ropivacaine 0.75 mg/ml (28 ml), epinephrine 1 mg/ml (0.5 ml), and ketorolac 30 mg/ml (1 ml) added to 50 ml of normal saline to make 80 ml of solution. Fifty patients were chosen and randomly divided into two groups of 25 each by computer-generated randomization. The case group received the cocktail injection, and the control group was injected locally with normal saline. Visual analog scale (VAS) was assessed at 3, 6, 12, and 24 hr post-surgery, and the amount of additional analgesics used and ambulation time were assessed.

Results: A total of 50 patients who underwent TKA were selected and divided into case and control groups of 25 each. The majority of the patients had osteoarthritis, and a few had rheumatoid arthritis. No significant differences in demographic data (age, gender, body-mass index) or surgical time. The case group had excellent VAS scores between 0 and 3 at 3, 6, 12, and 24 hr (p < .001). The amount of additional analgesia required in the case group was minimal (<3 doses) in 96% of the patients on Day 1. More than 80% of patients could ambulate pain-free on Day 1.

Conclusion: Pain reduction with early ambulation was noted in a significant number of individuals with the use of this unique intraoperative local cocktail injection. This pain-free initial period following TKA prepared patients for an effective rehabilitation program.

## Introduction

For patients with advanced knee osteoarthritis, total knee replacement (TKR) is one of the most frequently performed replacement surgeries in the orthopedic department [1]. Early postoperative pain, however, has remained unaddressed. In the first 36 hr after TKR, 60% of patients experience severe postoperative pain, and 30% experience moderate pain. Many patients opted out of the surgery for fear of postoperative pain [2].

Postoperative rehabilitation and physiotherapy, which are essential for maintaining the range of motion in the joint, will be hampered if adequate and effective postoperative analgesia is not provided [3]. Regional nerve blocks, systemic opioids, and epidural blocks are some of the pre-, intra-, or postoperative options. Each has its own risks and benefits. While systemic opioids offer superior pain control, they result in nausea, vomiting, sedation, and constipation; epidural infusions with or without an opioid are associated with vascular issues, urinary problems, and difficulty in ambulation [4,5]. In addition, epidural anesthesia is associated with hematoma formation if the patient is under thromboprophylaxis [6].

An appealing possibility is to administer local analgesia for surgical trauma with little risk of systemic side effects and without the need for expertise. It has been shown that intra-articular injections of various analgesics during TKR decrease the requirement for postoperative painkillers and also hasten hospital discharge.

Many studies have investigated the use of various combinations of analgesics administered intra-articularly during TKR, including local anesthetics, steroids, and nonsteroidal anti-inflammatory drugs (NSAIDs). The current study examines the effectiveness of an intraoperative periarticular local injection of multimodal drugs, including an NSAID (ketorolac), a long-acting local anesthetic (ropivacaine), and adrenaline, to provide analgesia following total knee arthroplasty for effective and early rehabilitation.

## Materials and methods

Study protocol

The study was a single-center, parallel-group, participant-randomized, case-control study of the effect of a cocktail of drugs given intraoperatively and periarticularly during TKR. Fifty patients were randomly divided into two groups of 25 according to a computer-generated sequence in the orthopedics department at Bangalore Medical College and Research Institute. Group A served as the case group, and Group B served as the control group. All the patients were given information regarding the visual analog score (VAS). Institutional review board (BMC/PGs/159/2015-16) consent was obtained.

Intervention

**Figure 1:**
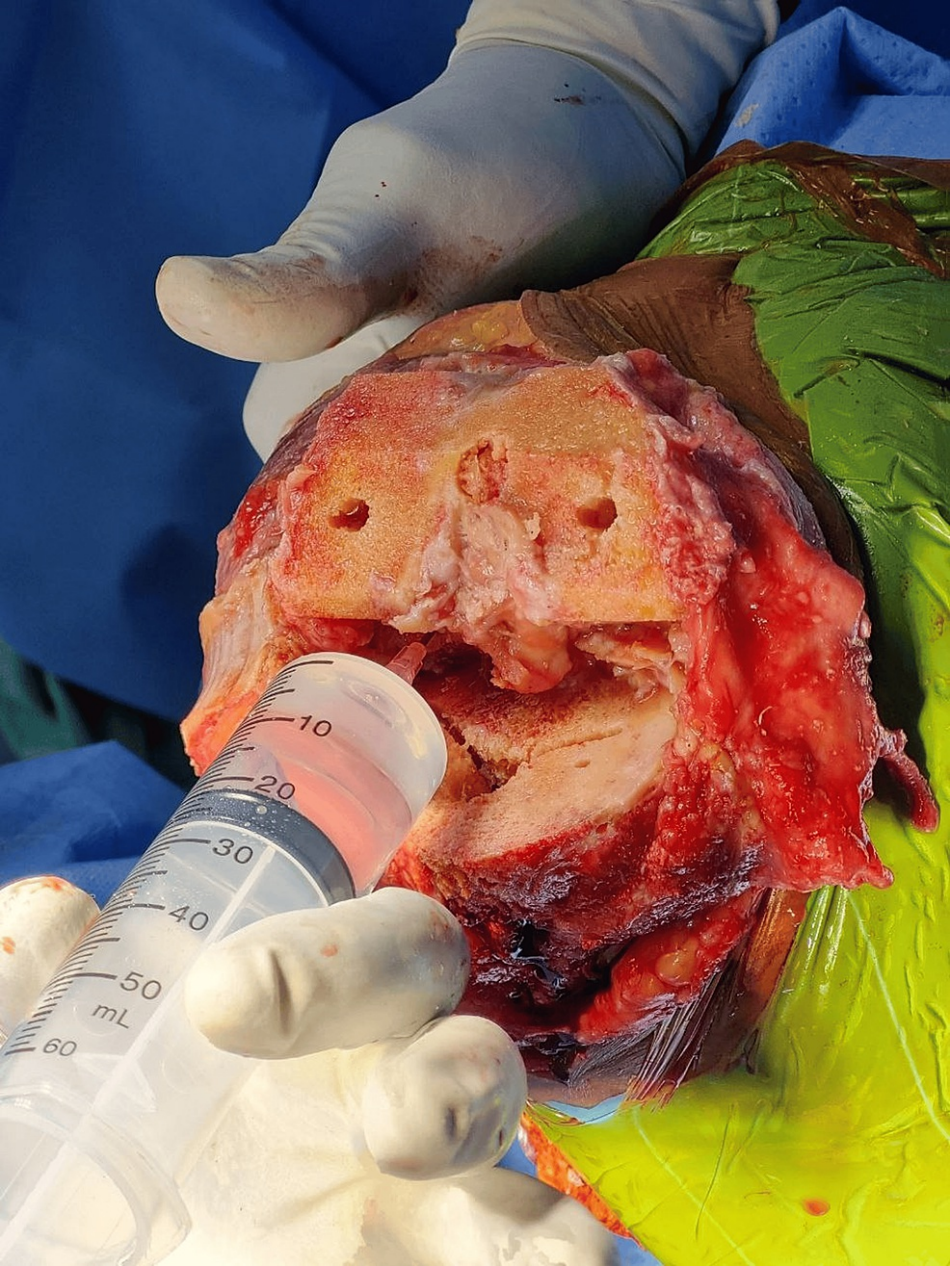
Cocktail infiltration into the knee's posterior capsule

**Figure 2:**
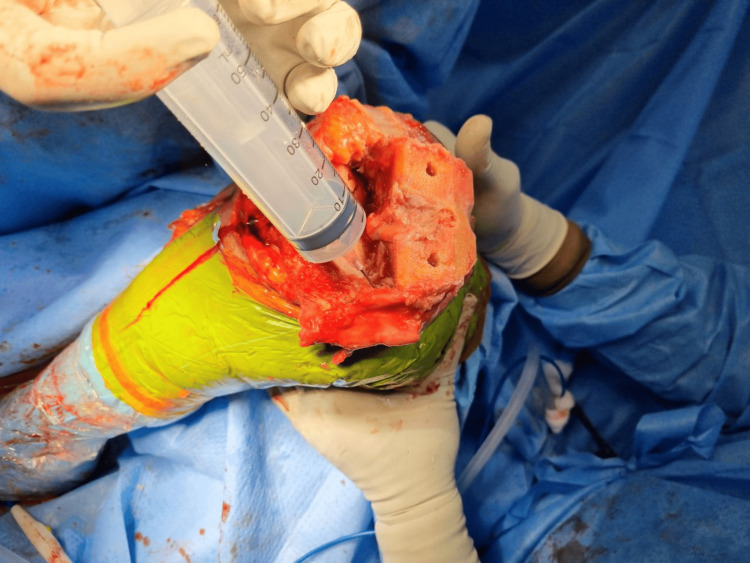
Cocktail infiltration into the medial periosteum, medial capsule, and medial collateral ligament

**Figure 3:**
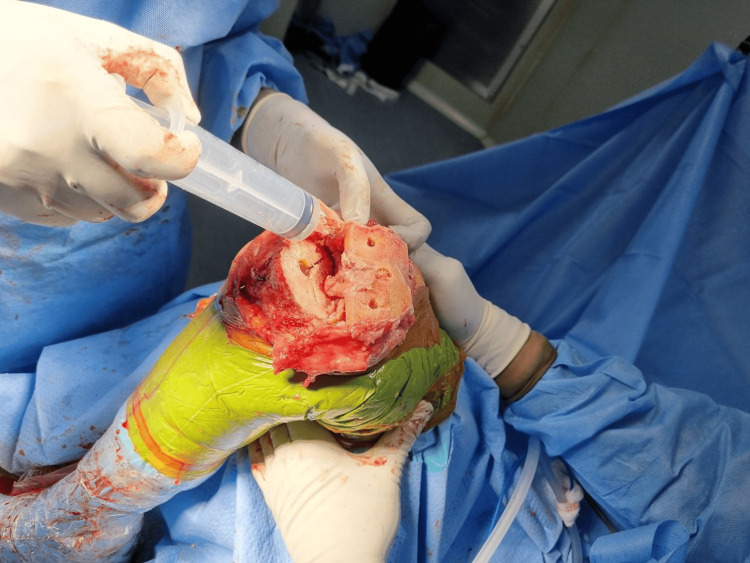
Cocktail infiltration into the lateral collateral ligament and the lateral meniscus capsular attachment

**Figure 4:**
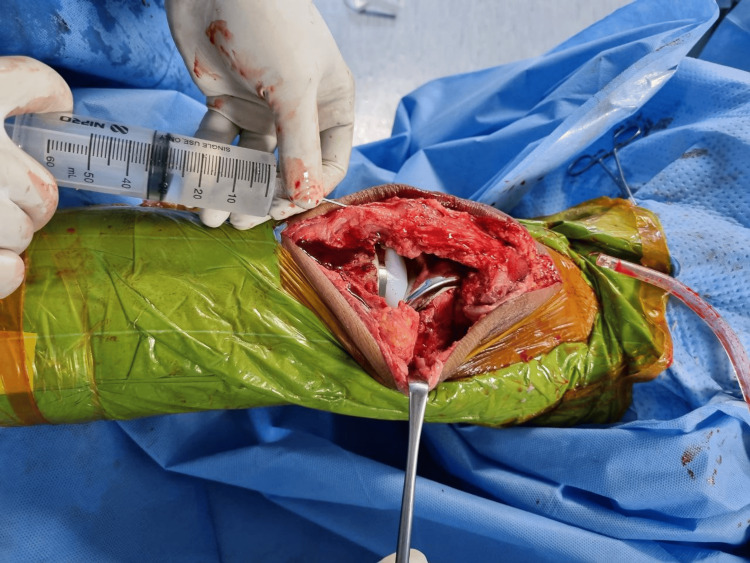
Cocktail infiltration into the patellar tendon and fat pad

A single surgeon performed all of the replacements under spinal anesthesia and a cementless cruciate retaining implant was used. A medial parapatellar approach was used under tourniquet control. Periarticular injection consisted of ropivacaine 0.75 mg/ml (28 ml), epinephrine 1 mg/ml (0.5 ml), and ketorolac 30 mg/ml (1 ml) added to 50 ml of normal saline to make an 80 ml solution. The cocktail was divided into quarters and injected into four zones: first, posterior periosteum and posterior capsule, including the posterolateral and posteromedial corners (Figure 1); second, medial periosteum, medial capsule, and medial collateral ligament attachments (Figure 2); third, lateral periosteum, lateral capsule, and lateral collateral ligament attachments (Figure 3); and fourth, anterior capsule, retinaculum, rectus tendon, patellar tendon, deep fascia, and subcutaneous tissue around the skin incision (Figure 4).

The cocktail was infiltrated before implant placement in the first, second, and third quarters, while the fourth quarter of the cocktail was infiltrated postimplant placement. While infiltrating the posterior structures, utmost care was taken to avoid inadvertent infiltration of neurovascular structures. Five minutes of fixation time were allowed for the drug to fix into the tissues after infiltration. The tourniquet was released after implant placement, hemostasis was achieved, and the wound was closed in layers without a drain.

Postoperative protocol

Postoperative thromboprophylaxis was started on the day of surgery, 6 hr after the surgery, and given in the form of low-molecular-weight heparin (enoxaparin 40 mg) to all patients. Routine analgesics in the form of paracetamol (1 g) infusion, tramadol (50 mg), and diclofenac sodium (75 mg) were given whenever additional analgesia was required, and the same was recorded.

Outcome measurement

Postoperative VAS was assessed at 3, 6, 12, and 24 hr post-surgery. The postoperative amount of analgesics used, ambulation time, and duration of hospital stay were assessed. On the first postoperative day, quadriceps and range-of-motion exercises began, and on the second postoperative day, protected weight-bearing ambulation began. In a few instances, however, weight-bearing was delayed because of discomfort.

Statistical analysis

The collected data were tabulated, coded, and analyzed using the Statistical Package for Social Sciences (SPSS), version 17.0 (SPSS Inc., Chicago, IL). At the end of the first month following surgery, the WOMAC score and the Knee Society Score were used to evaluate the functional outcome. Student’s t-test, chi-square, and Pearson coefficient tests were used to analyze the results.

## Results

Fifty pairs of knees were selected for the study, most of which were operated on for osteoarthritis and a few for rheumatoid arthritis. No significant differences between the two groups were found in demographic data (age, gender, body-mass index) or in surgical time.

Table 1 shows the VAS at 3, 6, 12, and 24 hr. Most of the patients (22) in the case group had VAS scores between 0 and 3, indicating excellent control of pain compared to the control group. Compared to the control group, a statistically significant reduction in pain was noted at 3, 6, 12, and 24 hr (p < .001).

**Table 1:** Comparison of VAS scores at different time intervals VAS: visual analog scale

VAS score	Case			Control			P value
Intensity of pain	0-3 Mild	4-6 Moderate	7-10 Severe	0-3 Mild	4-6 Moderate	7-10 Severe	
0-3 hr	22	2	1	2	23	0	<0.001
3-6 hr	11	6	8	0	5	20	<0.001
6-12 hr	15	8	2	0	18	7	<0.001
12-24 hr	15	6	4	0	19	6	<0.001

The amount of additional analgesia required in the case group was minimal (<3 doses) in 96% of the patients on Day 1. Compared to the control group, there was a statistically significant reduction in the amount of additional analgesia required (p < .001) on Days 1 and 2, as shown in Table 2.

**Table 2:** Comparison of the amount of additional analgesia on different days

Amount of additional analgesia	Case		Control		P value
	<3 doses	4-6 doses	<3 doses	4-6 doses	
Day 0	24	1	15	10	<0.001
Day 1	24	1	7	18	<0.001
Day 2	25	0	9	16	<0.001
Day 3	25	0	24	1	0.312
Day 4	25	0	25	0	

Ambulation was started on Day 1 post-surgery, and 20 (80%) patients were able to ambulate on Day 1, compared to 10 (40%) in the control group. Ambulation time was significantly lower in the case group compared to the control group, and the difference was statistically significant (p < .013), as shown in Table 3.

**Table 3:** Comparison of ambulation time on different days

Ambulation time	Case	Control	P value
Day 1	20	10	<0.013
Day 2	4	12	<0.013
Day 3	1	3	<0.013

## Discussion

TKA is a common orthopedic procedure, involving extensive tissue dissection. The resulting postoperative pain is a complicating factor for patients’ early rehabilitation. Delays in physical therapy and early rehabilitation lead to longer hospital stays, more medical expenses, and a greater load on the healthcare provider [7-8]. Multimodal analgesia has become routine in many centers performing TKA due to its effectiveness in counteracting initial postoperative pain with minimal side effects. Various combinations of a local anesthetic, an opioid, and an NSAID with epinephrine are used for postoperative pain management.

The active ingredients of our study include ropivacaine 0.75 mg/ml (28 ml), epinephrine 1 mg/ml (0.5 ml), and ketorolac 30 mg/ml (1 ml) added to 50 ml of normal saline. Most previous studies used bupivacaine as one of the components of the cocktail. Even though ropivacaine shares some pharmacokinetic characteristics with bupivacaine, it has a longer half-life, lower toxicity to the heart and central nervous system, and a higher tolerability in patients [9-10]. After the injection, the maximal circulation level is attained in about 20 to 30 min. Although blocking afferent peripheral nociceptive activity is ropivacaine’s primary mechanism of action, the medication has also been demonstrated to have some anti-inflammatory actions in human mucosal cells [11]. The second component of the cocktail, epinephrine, is thought to lower the anesthetic toxicity and keep the drug localized to the injection site [12]. The third component, ketorolac, works as an anti-inflammatory and analgesic and also augments the activity of other drugs when given along with other oral NSAIDs, thereby reducing the requirement for these systemic agents [13].

Our prospective randomized clinical study found that, compared to the control group, for the first 24 hr after the procedure, the VAS showed a significant reduction in pain (p < .001). This finding is similar to those of other studies. Essving et al., for example, examined the effect of a cocktail containing ropivacaine, ketorolac, and epinephrine [12]. Their study concluded with excellent VAS scores and statistically significant postoperative data at 3, 6, 12, and 24 hr. Moreover, in a study by Fu et al., the trial group’s VAS at rest was considerably lower than the control group’s at 6, 10, 24, and 36 hr following surgery [14]. Furthermore, Busch et al. concluded that the combination cocktail injection of ropivacaine, ketorolac, epimorphine, and epinephrine resulted in less PCA during the first 24 hr post-surgery [15]. Additionally, Anderson et al. compared periarticular cocktail injection with epidural infusion and found a statistically significant VAS score in the group infiltrated with the cocktail compared to the group infused with the epidural [16]. In their study of morphine use during the active treatment period (0-48 hr), Anderson et al. discovered a statistically significant decrease in the need for further analgesia [16].

In our study, the amount of additional analgesia in the form of either an NSAID or opioid was minimal (<3 doses) in most of the patients on Day 1. This is in agreement with the study by Kerr and Kohan, in which individuals who received the cocktail infiltration used fewer analgesics at 6 hr (p < .001) and 12 hr (p < .001) and required significantly lower analgesics [17].

Regarding ambulation, more than 80% of the patients in our study were able to ambulate on Day 1, compared to just 40% of the patients in the control group. Postoperative pain was the predominant reason for the patients in the control group not starting ambulation on Day 1. A study by Martin et al. reported similar findings that ambulation was significantly improved with the use of intraoperative cocktail injections [18].

Our study is limited, however, in that the concentration of each constituent in the mixture cannot be precisely determined. Moreover, although it was not possible to assess it, the normal saline that was injected into the control group may also be a factor that causes pain.

## Conclusions

The results of our study demonstrate a unique combination of drugs given locally that not only reduces postoperative pain but also decreases the amount of additional analgesia and brings forward the beginning of ambulation. This combination of drugs without an opioid allows for early rehabilitation, which is the cornerstone of a successful total knee arthroplasty. However, further studies are necessary that focus on the optimal quantities and combinations to achieve a pain-free ambulated patient. 
